# Alignment of protein structures in the presence of domain motions

**DOI:** 10.1186/1471-2105-9-352

**Published:** 2008-08-27

**Authors:** Roberto Mosca, Barbara Brannetti, Thomas R Schneider

**Affiliations:** 1 IFOM, the FIRC Institute for Molecular Oncology Foundation, Via Adamello 16, 20139, Milan, Italy; 2 European Institute of Oncology, Via Ripamonti 435, 20141, Milan, Italy; 3 European Molecular Biology Laboratory Hamburg Outstation c/o DESY, Notkestraße 85, 22607, Hamburg, Germany; 4 Novartis Pharma AG, CH-4056 Basel, Switzerland

## Abstract

**Background:**

Structural alignment is an important step in protein comparison. Well-established methods exist for solving this problem under the assumption that the structures under comparison are considered as rigid bodies. However, proteins are flexible entities often undergoing movements that alter the positions of domains or subdomains with respect to each other. Such movements can impede the identification of structural equivalences when rigid aligners are used.

**Results:**

We introduce a new method called RAPIDO (Rapid Alignment of Proteins in terms of Domains) for the three-dimensional alignment of protein structures in the presence of conformational changes. The flexible aligner is coupled to a genetic algorithm for the identification of structurally conserved regions. RAPIDO is capable of aligning protein structures in the presence of large conformational changes. Structurally conserved regions are reliably detected even if they are discontinuous in sequence but continuous in space and can be used for superpositions revealing subtle differences.

**Conclusion:**

RAPIDO is more sensitive than other flexible aligners when applied to cases of closely homologues proteins undergoing large conformational changes. When applied to a set of kinase structures it is able to detect similarities that are missed by other alignment algorithms. The algorithm is sufficiently fast to be applied to the comparison of large sets of protein structures.

## Background

When comparing structures of related proteins with different amino-acid sequences it is necessary to first perform a structural alignment, i.e. to define an equivalence map between the residues in the different structures based on their relative position in space. Once structures have been successfully aligned in three dimensions, similarities and differences can be studied in order to understand function and behaviour of the molecules under consideration.

It has been demonstrated that the problem of defining an equivalence map for residues in protein structures has no unique optimal solution [1] and that it remains computationally hard [2-4] even when it is described by a well defined optimization function. Nevertheless, many tools have been created for the pairwise and the multiple alignment of protein structures using different heuristics to produce results on acceptable time-scales (for comprehensive reviews see [5-7]).

Alignment methods can be classified based on whether the two structures to be aligned are considered as rigid bodies or whether internal flexibility between domains or subdomains is accommodated in the alignment. Methods belonging to the group of 'rigid aligners' are SSAP [8], CE [9], ProSup [10], KENOBI [11], MAMMOTH [12], TOPOFIT [13], TM-align [14], SABERTOOTH [15] and TetraDA [16]. DALI [17] allows for limited molecular flexibility through the use of an elasticity term in its similarity function, but nevertheless is considered to be a rigid aligner [18]. The group of rigid aligners also includes algorithms like VAST [19] and SSM [20] that, in order to produce alignments rapidly, first identify correspondences between secondary structure elements (SSE) and then extend the alignment to the residue level. Several rigid aligners have been extended for addressing the multiple alignment problem (CE-MC [21] and MAMMOTH-Mult [22]).

As it is well known, protein molecules are flexible entities with internal movements ranging from the displacement of individual atoms to movements of entire domains or subdomains [23,24]. Large-scale movements of groups of atoms complicate the correct identification of structural equivalences between related proteins when rigid structural aligners are used.

The molecular chaperon GroEL is an interesting case of protein molecules exhibiting pronounced molecular flexibility between structurally conserved domains. By comparison of crystal structures of different functional states, the GroEL molecule can be divided into three domains (equatorial, hinge and apical) separated by hinge regions [25]. Due to the large relative motion of the domains between different functional states, rigid body aligners will typically fail to align crystal structures of GroEL with different sequences in different conformational states.

In recent years, tools for the flexible alignment of protein structures have been introduced. These tools find an equivalence map between the residues of two molecular structures even when substantial intramolecular movements occur around molecular hinges. The regions between hinge points are commonly considered as rigid bodies and the alignment is usually optimized to minimize the number of hinges. The group of 'flexible aligners' includes, FlexProt [26] and FATCAT [18] and their corresponding extensions to multiple alignment MultiProt [27] and POSA [28].

**Figure 1:**
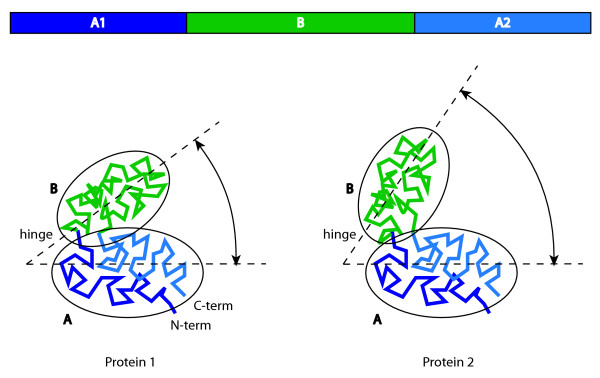
**Alignment of two proteins with a conformational change and a polypeptide chain folding back onto itself**. For two hypothetical proteins with homologous structures (protein 1 and protein 2), with two domains (one consisting of stretches A1 and A2 and one consisting of stretch B in sequence space) moving with respect to one another around a hinge, the aligned parts of the sequence are shown at the top, while the mapping of the alignment onto structures is shown with the same colours in the bottom of the figure. The alignment of proteins of such topology (e.g. GroEL) poses two problems: (1) the treatment of large conformational changes involving the motion of domains around hinge-regions (closed form of protein 1 versus open form of protein 2) and (2) the recognition of domains that are continuous in space but discontinuous in sequence (domain A of protein 1 and protein 2 consisting of parts of the N- (A1) and C-termini (A2)).

However, in alignments of molecules such as GroEL where the polypeptide chain folds back onto itself (Figure 1) and thereby creates structural domains in which parts of the polypeptide chain that are distant in sequence space engage into stable contact in three-dimensional space (e.g. for the equatorial domain of GroEL, see below), many of these aligners meet difficulties in recognizing the spatial continuity as will be illustrated below.

Here we introduce a new algorithm for the flexible structural alignment of proteins called RAPIDO (for Rapid Alignment of Proteins in terms of Domains). RAPIDO is capable of aligning related protein molecules in the presence of large conformational differences while at the same time groups of equivalent parts of the polypeptide that are distant in sequence but nevertheless form spatially continuous domains are identified correctly as such. As a first step RAPIDO creates an equivalence map between the two structures by taking into account flexibility, with a procedure that is similar to the one used by FATCAT [18]. This step is followed by the application of a genetic algorithm [29] for the identification of *structurally conserved regions *that can be continuous in space but not in sequence (e.g. the equatorial domain of GroEL). The result of the procedure is a description of a protein in terms of structurally conserved regions connected by localized hinges or by flexible linker regions. We have chosen the standard parameter settings for RAPIDO such that more emphasis is placed on the geometric similarity of the structurally conserved regions (as reflected in low RMSDs) than on their size (as reflected in the length of the alignments). With this choice, the resulting structurally conserved regions will have a high level of similarity allowing their usage for robust coordinate-based structure superpositions.

In the following, we describe the algorithm used and the application of RAPIDO to a number of test cases. For all test cases, RAPIDO produces results that are in agreement with previous analyses. Regions identified as structurally conserved furnish subsets of atoms whose relative positions between different structures are very well maintained. Superpositions based on these subsets of atoms are particularly revealing when molecular flexibility is studied.

## Results

### Algorithm

The alignment algorithm consists of four steps:

1. Search of short structurally similar fragments in pairs of structures, so called Matching Fragment Pairs (MFPs)

2. Chaining of the MFPs through a graph-based algorithm

3. Refinement of the alignment

4. Identification of rigid bodies

In the remainder of this section we will refer to two structures being compared as structures A and B. The *i*-th residue in structure *X *(*X *= A or *X *= B) is represented by the coordinates of its C_*α *_atom and will be indicated by **x**_*i *_(**a**_*i *_and **b**_*i *_respectively).

### Finding matching fragments

We define a *fragment *as an ungapped stretch of residues and a *matching fragments pair *(MFP) as a pair of structurally similar fragments of the same length in two structures being compared. The search for MFPs is in fact implemented in a number of alignment tools as the initial step [9,10,18,26,30] because it significantly reduces the complexity of the search space for the alignment. Pairs of similar fragments named *matching fragment pairs *(MFPs) here, have been named *aligned fragment pairs *(AFPs) in other publications [9,18,30]. In the context of the RAPIDO aligner, we prefer to use the notation of *matching fragment pairs *in order to clarify that in a later stage of the alignment algorithm, a subset of the *matching fragment pairs *forming the initial set is selected to assemble the actual alignment, and the selected MFPs thus become *aligned fragment pairs*.

While many algorithms use the RMSD to measure the similarity between two fragments [18,26,30], we use an alternative measure, the sum of the absolute values of the elements of the difference distance matrix between the C_*α*_-atoms of the two fragments (eq. 1 in the *Methods *section).

At first an exhaustive search for MFPs of length *m*_*L *_(*m*_*L *_= 8 in the implementation) is performed, followed by a clustering step in which overlapping MFPs are joined to form longer ones.

### Chaining matching fragment pairs and refining the alignment

The MFPs identified in the first step constitute a set of potential building blocks for the final alignment from which, in the second step, a subset of MFPs representing a structural alignment is assembled. This is done by casting the problem into a graph representation to which a standard algorithm for identification of the longest path is applied. The MFPs are represented as vertices of a graph and two MFPs (e.g. two vertices) are connected by an edge if they are topologically ordered, i.e. if they are composed of two pairs of fragments that appear in the same order in the two residue sequences. Every path in the graph represents a possible alignment and by choosing an appropriate *weight function *for the edges, the problem of finding the best alignment is translated into the problem of identifying the longest path on a graph. We solve this problem by applying a dynamic programming algorithm for the identification of the longest path. The alignment obtained in this way is a preliminary alignment that is then refined (details on the refinement process can be found in the *Methods *section) resulting in the *raw alignment*.

### Identification of rigid bodies and flexible superposition

Once the raw alignment has been calculated, the algorithm performs a search for structurally conserved regions. Structurally conserved regions relate to conformationally invariant regions detected in different conformations of the same molecule as described in [31]. Conformationally invariant regions can be defined as subsets of equivalent atoms whose interatomic distances are identical within error between the different conformations of the same molecule [31]. In the comparison of different molecules, the concept can be generalized by considering subsets of *aligned *residues for which distances between C_*α*_-atoms are identical within a tolerance as *structurally conserved regions*. These subsets can be identified using a genetic algorithm operating on scaled difference distance matrices [29,31,32]. In our previous work the elements of the difference distance matrix were scaled by propagated coordinate errors resulting in error-scaled difference distance matrices [31]. The parameters necessary for the estimation of the coordinate errors were extracted automatically from the PDB files and if necessary corrected manually. This approach is not applicable when very many PDB-files are being investigated in the context of searching for related structures in large data bases as the values extracted can be unreliable mostly caused by human errors made when the parameters where entered in the first place. For the purpose of structural alignment, we therefore use a simplified approach in which the estimate for the coordinate error of an atom *i *with a B-value of *B*_*i *_is replaced by an analogous quantity ${\tilde{\sigma }}_{i}$ calculated as follows

(2) $${\tilde{\sigma }}_{i}=k\cdot {\left(1+\frac{B_{i}}{2\pi^{2}}\right)}^{\eta },$$

where the constants *k *and *η *have been empirically optimized to 0.4 Å and 2/3. ${\tilde{\sigma }}_{i}$ can then be propagated into a scaling-factor for difference distance matrix elements in a manner similar to the previous treatment.

The algorithm searches iteratively for structurally conserved regions in analogy to the approach presented in [32]). Aligned residues that cannot be assigned to structurally conserved regions are marked as flexible.

To characterize the agreement between two structures after the equivalent residues have been divided into structurally conserved and flexible regions, separate least-square superpositions are performed for the different structurally conserved regions.

Based on this superposition allowing flexibility between conserved parts of a three-dimensional structure, we define the 'flexible RMSD' (*RMSD*_*f*_) as the standard RMSD calculated for all pairs of equivalent C_*α*_-atoms after separate least-squares superposition for the different structurally conserved regions.

### Testing

In order to assess the functionality of the method, we applied it to various test cases. Here, we describe the analysis of two structures of different topologies with known hinge-motions (Ran and GROEL) and we compare the results of RAPIDO with those obtained by FATCAT [18] and FlexProt [26]. Second, we compare the results obtained with RAPIDO with those given by DALI for 2278 pairwise alignments between 68 crystal structures of protein kinases from human.

### Ran

Ran is a small GTPase belonging to the Ras superfamily that plays an important role in several nuclear functions, including nucleocytoplasmic transport, cell-cycle progression and nuclear envelope assembly [33]. Here we compare two structures of Ran proteins from two different organisms: the first one is the structure of a Q69L mutant of Ran from dog with a bound GDP molecule (RanQ69L*GDP, PDB id 1byu, [33]); the second structure corresponds to Ran from human in complex with human RanBP2 and a non-hydrolysable GTP analogue (Ran*GppNHp complex, PDB id 1rrp, [34]).

**Figure 2:**
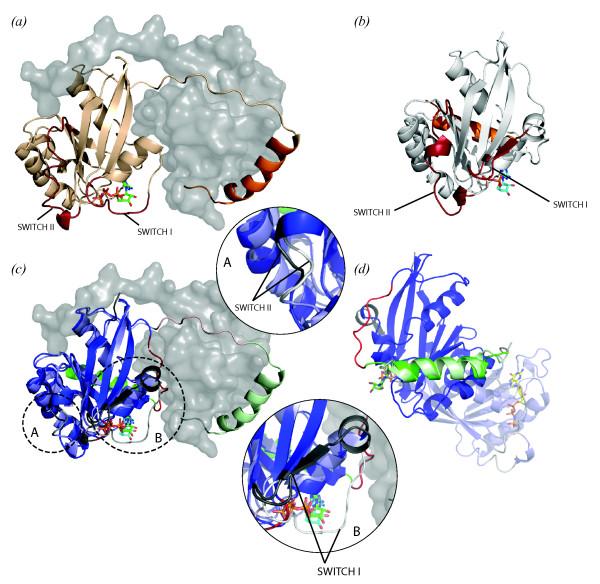
**Alignment of structures of two Ran proteins**. (a) Structure of human Ran (cartoon) bound to a non-hydrolysable GTP analogue (sticks) with the Ran-binding domain of human RanBP2 (grey surface). The SWITCH I and II loops are shown in red, the C-terminal helix is displayed in orange. (b) Structure of a Q69L mutant of canine Ran (cartoon) with a bound GDP molecule (sticks) (c) Superposition of the two Ran molecules on the first rigid body identified by RAPIDO (140 atoms, RMSD 0.76 Å). The different conformations of the SWITCH I and II fragments as well as the large displacement of the C-terminal helix are clearly visible. In this figure (and in all other figures), the first rigid body is colored in blue, the second in green, the third in cyan, the fourth in magenta. Parts of structures that cannot be aligned are marked in grey. Parts of structures that were aligned and then identified as having different conformations in different structures are colored red. When two structures are compared, one is shown in light, the other in dark colors – here the structure of the protein from human is shown in dark colors, while the structure from dog is shown in light colors. (d) Superposition of the two Ran molecules on the second rigid body consisting mostly of the C-terminal helix (in green, 18 atoms, RMSD 1.35 Å). The unstructured linker preceding the C-terminal helix has been found to be flexible and is marked red. All figures were produced with PyMOL .

The RAPIDO alignment shows that major parts of the two structures are very similar. 182 residues are aligned, 158 of which are assigned to two rigid bodies. The first rigid body covers more than 70% (140 residues) of the entire protein, can be superposed with an RMSD of 0.76 Å (Figure 2) and corresponds to the main body of the protein. Two fragments in this region are either not aligned or aligned but marked as flexible. They correspond to the well-known SWITCH I and SWITCH II regions, which exhibit different conformations depending on the type of bound nucleotide and regulate the interactions of the protein with nuclear trafficking components [35]. The C-terminal regions of the two structures have been aligned although they are located in very different positions with respect to the main body of the protein in the two structures. This region is composed of an unstructured loop followed by a helix that is known to assume a different conformation depending to the GTP/GDP-binding state of the protein [36]. The C-terminal helix is attached to the main body of the protein in the Ran*GDP complex while in the Ran*GppNHp complex, it interacts with a groove on the surface of the RanBD1-domain approximately 25 Å distant from the Ran main body. While the helix is recognized as a second rigid body, the part of Ran connecting its main body with the C-terminal helix in different conformations is marked as a flexible region.

The alignments between the two structures as produced by FATCAT and FlexProt are slightly longer (186 aligned residues for FATCAT, 188 for FlexProt). The separation between the two rigid bodies is similar in the three alignments but the RMSD for the superposition of the single rigid regions is higher in FATCAT and FlexProt alignments than in the RAPIDO alignment. This is due to the fact that in these two aligners all aligned residues are used for the superposition while RAPIDO distinguishes between structurally conserved and flexible aligned residues and uses only the residues in structurally conserved regions to perform the superposition. In fact, in the FATCAT and FlexProt align fragments the SWITCH I and II loops are attributed to the first equivalent region yielding an RMSD for the superposition of this first rigid part of 1.51 Å for FATCAT and 2.87 Å for FlexProt. The unstructured loop connecting the main body and the C-terminal helix is partly assigned to the first equivalent region and partly to the second adding to the increased RMSD-values for the respective superpositions.

Although, for this case, the alignments are mostly equivalent, the one provided by RAPIDO highlights the different conformations of three important functional elements corresponding to the SWITCH I and II loops and to the C-terminal loop and produces an accurate superposition of the two structures in which these differences can be clearly analyzed.

### GroEL

**Figure 3:**
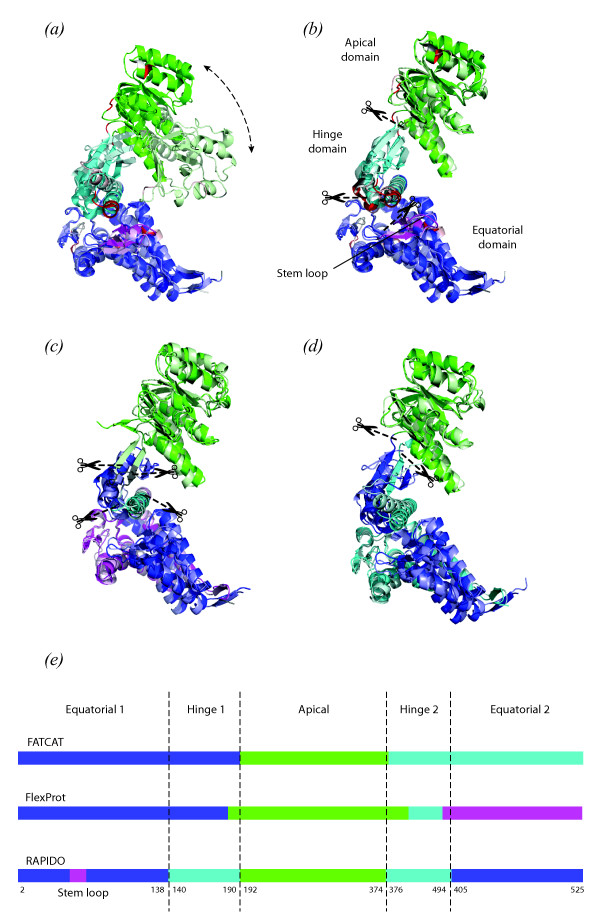
**Alignment of two structures of GroEL from *Thermus Thermophilus ***(1we3) **and *Escherichia Coli ***(1oel). (a) Superposition of the two structures on the first rigid body identified by RAPIDO (in blue, 1we3 is in darker colors while 1oel is in lighter colors). (b) Flexible superposition based on the rigid bodies identified by RAPIDO. Scissor symbols indicate the points in which the 1oel was divided in order to separately superpose the regions identified as rigid bodies (1^st ^rigid body: 220 atoms, RMSD 0.81 Å; 2^nd ^rigid body: 178 atoms, RMSD 0.93 Å; 3^rd ^rigid body: 71 atoms, RMSD 1.04 Å; 4^th ^rigid body: 20 atoms, RMSD 0.68 Å). (c) Flexible superposition generated by FlexProt (1^st ^fragment: 122 atoms, RMSD 2.62 Å; 2^nd ^fragment: 21 atoms, RMSD 3.02 Å; 3^rd ^fragment: 193 atoms, RMSD 2.95 Å; 4^th ^fragment: 177 atoms, RMSD 2.95 Å). (d) Flexible superposition generated by FATCAT (1^st ^fragment: 186 atoms, RMSD 3.17 Å; 2^nd ^fragment: 179 atoms, RMSD 0.96 Å; 3^rd ^fragment: 153 atoms, RMSD 3.17 Å). (e) Mapping of the conserved domains identified by different methods onto the primary sequence. Residue numbers of domain boundaries in the *E. Coli *structure (1oel) as determined by RAPIDO are indicated; small flexible insertions within the domains have been left out for clarity.

GroEL is a bacterial chaperonin that, together with its co-chaperonin GroES forms a system helping newly synthesized polypeptides to reach their native state in the crowded cellular environment. GroEL consists of 14 identical subunits that are assembled as two heptameric rings stacked back to back, forming a cavity in the centre in which a newly formed polypeptide can find a protected environment for refolding [37]. Each subunit corresponds to a single protein molecule with three domains called the equatorial, apical and hinge domain (Figure 3b). During its activity, the GroEL complex undergoes dramatic conformational changes correlated with different relative arrangements of the three domains in each subunit. Here we align the structure of one GroEL subunit from *Escherichia coli *(PDB id 1OEL, [38]) with one from *Thermus termophilus *in complex with ADP (PDB id 1WE3, [39]).

The structural alignment produced by RAPIDO covers 98% of the molecule (516 aligned residues), with a flexible RMSD of 0.88 Å. Four structurally conserved regions are identified (Figures 3b and 3e) corresponding to the three canonical domains of the GroEL subunit plus the stem loop in the equatorial domain comprising approximately 20 residues. The three structurally conserved regions are in different relative positions with respect to each other in the two structures as highlighted by the RMSD of 11.59 Å for the rigid superposition. However, by examining the superposition of the structurally conserved regions separately, the structural conservation of major parts of GroEL can be well appreciated both from the RMSDs ranging between 0.81 and 1.04 Å and the actual superposition (Figure 3). In addition to the three large canonical domains, the so-called stem loop in the equatorial domain is found to constitute a small structurally conserved region assuming different orientations in the two structures. This dependence of the positions of the stem loop on the functional state had already been observed by Xu et al. [25].

The alignment produced by FATCAT has approximately the same length (518 residues) and a flexible RMSD of 2.45 Å. Two hinges are identified and the structure is divided into the three regions shown in Figure 3d. While the apical domain is identified by both RAPIDO and FATCAT as an equivalent region, the equivalent regions for the other two domains display marked differences. The hinge domain is, in the FATCAT alignment, joined to the equatorial domain and the resulting superposition is thus an average between the superposition of the two single subunits, leading to a higher value for the RMSD. Due to the sequential constraint imposed by FATCAT (two regions that are not sequential cannot belong to the same rigid body), the block corresponding to the equatorial-hinge domain is split into two fragments corresponding to the N- and C-terminal parts. The stem loop is in the FATCAT alignment included in the first rigid region.

FlexProt creates an alignment of 513 residues with a flexible RMSD of 2.87 Å. Three hinge-points dividing the structure in four fragments are identified. As in the FATCAT alignment, the apical domain is kept separate from the rest of the structure. Even if the C-terminal parts of the hinge and equatorial domains are separated by a hinge point, their N-terminal counterparts are kept together including the stem loop. In general, the alignments produced by FATCAT and FlexProt tend to underestimate the number of hinges for this pair of structures and cannot be used to highlight the difference between the equatorial and hinge domains, nor the different conformation of the stem loop.

A correct delineation of the domains is of particular interest in this case. In fact, the identified domains can be used as rigid bodies for the interpretation of low-resolution electron density maps for GroEL in different functional states as determined by electron microscopy. In this way, they allow to derive conclusions at the atomic level from lower resolution data (e. g. Ranson et al. [40]).

### Human kinase structures

Protein kinases are multi-domain proteins catalyzing the phosphorylation of proteins and play important roles in controlling many cellular processes (chapter 13 in [41]). The protein kinase catalytic domain consists of two lobes, a small N-terminal lobe and a large C-terminal lobe connected by a hinge region and is often augmented by other domains that serve in regulation of the kinase activity. Prominent examples of such domains are the SH2 and SH3 domains present in protein kinases such as src Hck kinase [42] and Bcr-abl kinase [43]. In protein kinases, the relative positions and orientations of the different domains are very variable and depend on many factors such as the binding of ligands in the active site and/or the presence of regulating factors.

We used RAPIDO to perform an all-against-all alignment for 68 structures of human protein kinases (2278 alignments in total). For comparison, for every pair of structures, an alignment was also determined using DaliLite Ver. 2.4.4 (the standalone version of DALI).

**Figure 4:**
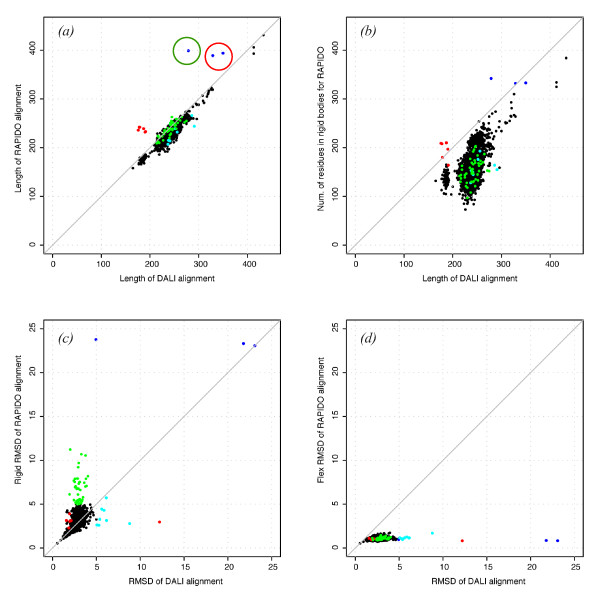
**Comparison between DALI and RAPIDO on the dataset of human kinase structures**. Every dot in the scatter plots represents one of the 2278 alignments between 68 structures (a) Length of the raw alignment provided by RAPIDO vs. the length of the corresponding DALI-alignment. Blue and red dots represent pairs of structures for which the RAPIDO alignment is significantly longer than the DALI alignment. Green and cyan dots indicated structures for which the rigid RMSD of the RAPIDO-alignment is substantially higher than that for the DALI-alignment or *vice versa *(Panel (c)). Data points surrounded by circles are discussed in the text. (b) Total number of residues assigned to rigid domains by RAPIDO vs. length of DALI alignment (c) Rigid RMSD for all atoms aligned by RAPIDO vs. rigid RMSD for atoms aligned by DALI. (d) Flexible RMSD for atom aligned and identified as belonging to rigid bodies by RAPIDO vs. rigid RMSD for all DALI-aligned atoms. Please note that the lengths and RMSDs given for the RAPIDO alignments correspond to *aligned *residues in Panels (a) and (c) while they correspond to *rigid *or *structurally conserved *residues in Panels (b) and (d); the difference between the two sets are *flexible *residues that have been aligned but are found in different conformations in the structures being compared.

Alignments produced by RAPIDO and DALI are compared in Figure 4 and summarized in additional file 1. In terms of overall length, the majority of the alignments are comparable. However, for some cases, the RAPIDO alignments are substantially longer than the DALI alignments (blue and red dots in Figure 4).

**Figure 5:**
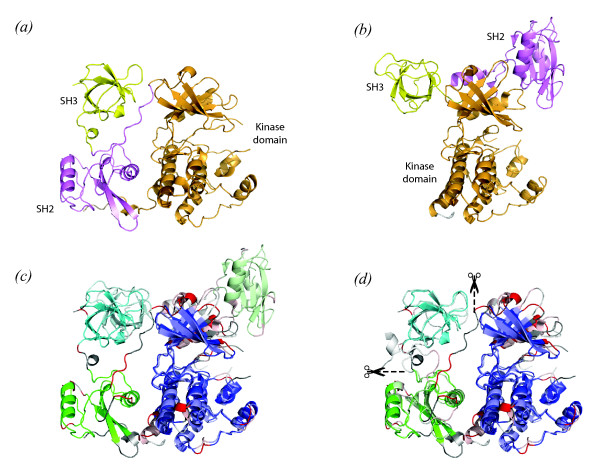
**Alignment of structures of Hck and Csk protein kinases**. Panel (a) and (b) show schematic drawings of the structures of Hck (PDB id 1ad5) and Csk (PDB id 1k9a) src kinases. The kinase domains, the SH2, and the SH3 domains are shown in orange, magenta, and yellow, respectively. (c) Superposition of both structures on the first rigid body, corresponding to the kinase domain (shown in in blue, 190 res, RMSD 0.90 Å). Hck kinase is shown in dark colors, Csk kinase in light colors. The substantially different positions of the SH2 and SH3 domains with respect to the kinase domain become visible. (d) Flexible superposition between the two structures. When superposed separately the three domains reveal a considerable level of structural conservation (1^st ^rigid body: 190 atoms, RMSD 0.90 Å; 2^nd ^rigid body: 81 atoms, RMSD 0.88 Å; 3^rd ^rigid body: 55 atoms, RMSD 1.06 Å).

Three of these cases (blue dots in Figure 4a) correspond to alignments between the structures of Hck from Human (1AD5, [42]), c-Src from Human (1FMK, [44]), Csk from Rat (1K9A, [45]) and c-Abl from Mouse (1OPK, [43]). In these four structures, the kinase domain was crystallized in the presence of SH2 and SH3 domains. Depending on the functional state of the kinase, the SH2 and SH3 domains can be in substantially different positions with respect to the kinase domain. Such different positions can cause rigid aligners not to recognize all domains as similar. For the case of the alignments between Hck and Csk, and between c-Src and Csk (dots in the red circle in Figure 4a), DALI aligns only 329 and 350 residues respectively with the aligned residues being located in the protein kinase domain and in the SH2 domain. The SH3 domain is not included in the alignment. For the alignment between Csk and c-Abl (dot in the green circle in Figure 4a) DALI aligns only the protein kinase domain. The alignment produced by RAPIDO in all three cases is longer (389 to 399 residues) and comprises the kinase domain as well as the SH2 and SH3 domains (Figure 5).

To illustrate different positions of domains in protein kinase structures, Figure 5 shows the alignment between the structures of Hck (PDB id 1ad5) and Csk (PDB id 1k9a). Although the positions and orientations of the SH2 and SH3 domains with respect to the protein kinase domain are substantially different in the two structures (Figures 5a and 5b), RAPIDO manages to align the two structures for almost their entire length identifying three separate structurally conserved regions (Figure 5c). The largest structurally conserved region corresponds to the conserved core of the protein kinase domain, while the two smaller structurally conserved regions are the SH2 and the SH3 domain. Superposition on the conserved part of the protein kinase domain clearly reveals the different positions and orientations of the SH2 and SH3 regulatory domains with respect to the catalytic domain (Figure 5c). By superposing the three regions separately (Figure 5d) the structural conservation of the different domains in the two protein structures becomes clear and a flexible *RMSD*_*f *_of 0.86 Å on 332 residues indicates the close relation between equivalent domains in different protein.

Other cases for which the RAPIDO alignment assigns more equivalent atoms than the DALI alignment concern alignments of structures with large differences in the opening angles measured between the N- and the C-terminal lobe of the kinase domain (red dots in Figure 4). For the alignment between the structures of the protein kinase domains of CDK6 (1BI7), MAPK P38 (1P38), Src (1FMK), IGF1 receptor (1JQH), EGFR (1M17), HGFR (1R0P) and JAK3 (1YVJ), the algorithm implemented in DALI can cope with many cases of different relative domain orientation. However for the cases marked in Figure 4, the residues in the N-terminal domain are not aligned due to the large differences in opening angle between the lobes. In one of these cases (red point in Figure 4c, corresponding to the alignment between 1FMK and 1R0P), parts of the small lobe are in fact included in the alignment but at the cost of a very large RMSD between the equivalent atoms (12.20 Å for 190 atoms, Figures 4c and 4d). In all these cases, RAPIDO correctly determines the equivalences between atoms both for the C- and the N-terminal lobe, independently of their relative positions.

There are cases where the 'rigid RMSDs' measured for the superposition based on all atoms aligned by RAPIDO is substantially higher than the rigid RMSD for the corresponding DALI alignments although the alignments are of comparable length (green dots in Figures 4a and 4c). These are cases where taking into account flexibility in the RAPIDO algorithm results in an alignment including small fragments that are locally very similar but structurally not equivalent when their surrounding environment is considered. A typical situation of this kind is the erroneous alignment of periodical structural elements such as *α*-helices or *β*-strands with a shift in register. Such fragments are included in an alignment because they exhibit high local similarity and their different positions with respect to neighbouring structural elements is assumed to be due to conformational change. Although for the majority of cases, these situations are remedied, it is generally not possible to avoid them without an unacceptable loss in sensitivity. However, such incorrectly aligned fragments will not be included into structurally conserved regions as their positions in different structures are inconsistent and therefore such fragments will be marked as *aligned *but *flexible *– this is the reason for the number of residues assigned to rigid bodies by RAPIDO being usually smaller than the number of residues aligned by DALI (Figure 4b). When the flexible RMSD is calculated for all atoms assigned to structurally conserved regions (leaving out the aligned but flexible atoms), it is substantially lower than the standard RMSD calculated for the corresponding DALI alignments (Figure 4d) thus indicating the presence of similarities more clearly.

Finally, in some cases the alignment produced by DALI is longer than the one produced by RAPIDO (cyan dots in Figure 4). However, careful analysis reveals that in these cases, the DALI-alignments comprise some small fragments that are locally similar but when put in the context of their structural neighbours should actually not be considered as equivalent. The presence of such inconsistencies is also reflected in the higher values for the rigid RMSD when compared to the RAPIDO alignments (Figure 4c).

### Implementation

The algorithm has been implemented in C++. For academic use, executables for various platforms can be obtained from the corresponding author upon request. A web server for aligning structures using the RAPIDO-algorithm is available at .

Typical execution times with the inclusion of the pre-processing step (see *Methods *section for details) range from 0.5 sec to 1.5 s for pairs of structures between 200 and 400 residues. Without pre-processing, execution time ranges between 1.5 and 4 s for the same structures (CPU-times for iMac with an Intel Core 2 Duo processor at 2.4 GHz and 2 GB of memory running under MAC OS X version 10.4).

On output, the program generates different files. A textual representation of the alignment is generated in an HTML file. Different types of superpositions are available: rigid superposition on all aligned atoms, superpositions on individual rigid bodies and flexible superposition. The latter is obtained by subdividing the structures into pieces centred on the rigid bodies identified in the alignment procedure. The parts of the structures falling between the boundaries of two rigid bodies are moved together with the rigid body closest in sequence during the superposition.

The superposed structures with their modified coordinates are stored as PDB files. PyMOL- and RasMOL-scripts for displaying the superposed structures are generated by the program. All output information is consistently color-coded based on the rigid body assignments so that conformationally invariant parts can be easily inspected.

## Conclusion

In this paper, we have introduced a new method named RAPIDO for the alignment of proteins in the presence of conformational changes. Aligned residues are grouped into subsets that can be considered as rigid domains with respect to the structures being compared; aligned residues not assigned to a rigid domain are considered flexible.

When applied to structures with known hinge motions, RAPIDO produces results that are consistent with manual analyses presented in the literature. By using a genetic algorithm operating on scaled difference distance matrices [29], structurally conserved regions are assembled consistently even when composed of fragments that are not continuous with respect to the polypeptide chain.

With standard settings, RAPIDO identifies subsets of residues whose C_*α*_-atoms can be superimposed with RMSDs of typically less than 1 Å for structurally conserved regions. Given the tight conditions in terms of similarity, the individual structurally conserved regions are generally smaller than those obtained by other alignment algorithms. However, as other regions that are in different relative positions in the structures under comparison will be aligned with high accuracy as part of different rigid bodies, the overall length of the combined alignment taking flexibility into account will be increased in many cases.

In the context of structure comparison and analysis, superpositions of structures based on atoms located in rigid domains can highlight conformational differences that, when superpositions are based on atoms sets accidentally containing flexible regions, can be difficult to identify.

The application of RAPIDO to a dataset of kinase structures showed how allowing for flexibility can help to detect similarities that are not found by rigid aligners.

To evaluate the limits of RAPIDO, we have applied the algorithm to ten 'difficult cases' of low sequence and structural similarity from Fischer's [46] dataset for benchmarking fold-recognition methods. The results obtained [see Additional file 2] indicate that for distantly related structures RAPIDO alignments are generally shorter and exhibit larger RMSDs than alignments produced by other algorithms. RAPIDO should therefore be used preferentially for cases were closely related structures are sought for.

A definite advantage of RAPIDO is the short time required to calculate an alignment. E.g., a total of 2278 alignments on a set of 68 kinase structures was completed by RAPIDO in 61 minutes. This allows applying the method presented to problems of substantial size such as querying a large set of structures for similarities with a structure of interest or all-against-all alignments of entries in structural databases.

## Methods

### Identification of matching fragment pairs

An MFP composed of two stretches of residues of length *L *starting at residue *i *in structure A and at residue *j *at structure B, is described by a triplet (*i*, *j*, *L*). A distance between the two fragments, S(*i*, *j*, *L*), is calculated as:

(1) $$S(i,j,L)=\frac{1}{L}\sum_{t=1}^{L-1}\sum_{s=0}^{t-1}\left|d_{A}(i+t,i+s)-d_{B}(j+t,j+s)\right|$$

where

*d*_*x*_(*u*, *v*) = ||**x**_*u *_- **x**_*v*_||

is the element of the distance matrix between the C_*α*_-atoms of residues *u *and *v *in structure *X*.

In the first step, the algorithm builds the list S* of MFPs of length greater than or equal to *m*_*L *_for which *S*(*i*, *j*, *L*) is lower than a threshold *m*_*S*_. Even if the number of possible fragments, $\sum_{L=m_{L}}^{M\equiv \min\{m,n\}}\left(n-L+1)(m-L+1\right)=O(M^{3})$, is polynomial in M, finding the complete set S* is computationally too expensive. To reduce the complexity of this step, we thus first search for all MFPs of fixed length *m*_*L *_and distance *S*(*i*, *j*, *L*) lower than a threshold *m*_*S*_. Then we identify groups of overlapping MFPs and test whether groups of MFPs can be merged into one larger MFP. If the score for the merged MFP is lower than the chosen threshold *m*_*S*_, it is kept. Technically, the merging step consists of extending a randomly chosen MFP downstream with overlapping MFPs until the score of the merged MFP becomes greater than the threshold *m*_*S*_. In the current implementation of the algorithm, *m*_*L *_= 8 and *m*_*S *_= 3.0.

### Chaining of matching fragments

**Figure 6:**
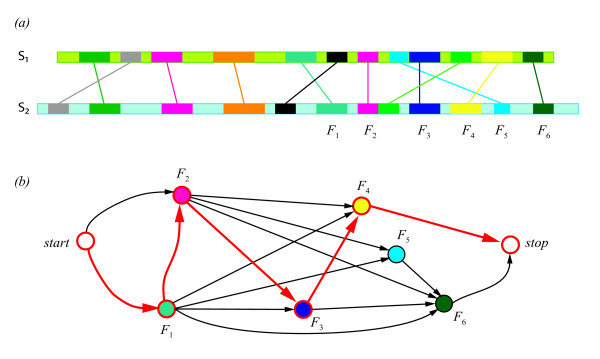
**Chaining of Matching Fragment Pairs**. A schematic representation of MFPs for two proteins with sequences S_1 _and S_2_. MFPs are indicated as pairs of rectangles connected by a line mapped onto the sequence in panel (a) and as nodes of a graph in corresponding colors in (b). The graph representation encodes the topological relations between the MFPs. E.g. *F*_3 _can be chained with *F*_6 _but it cannot be chained with *F*_5_, because *F*_5 _involves a fragment on sequence S_1 _that is upstream of the corresponding fragment of *F*_3 _on sequence S_1 _(Panel (a)). In the graph-representation, such a situation results in no edge assigned to the pair of vertices representing *F*_3 _and *F*_5_. By choosing an appropriate weight function for the edges (see text), the longest path corresponds to the best alignment between the two structures as represented here by thick red arrows.

In order to select the MFPs forming the alignment, first a graph representing all the MFPs identified in the first step of the algorithm is built. Every MFP becomes a vertex in the graph and two MFPs *F*_1 _and *F*_2 _are connected by an edge if they can be chained, i.e. if and only if *F*_1 _<<*F*_2 _according to the following definition (Figure 6):

*Let F*_1 _≡ (*i*_1_, *j*_1_, *L*_1_)*and F*_2 _≡ (*i*_2_, *j*_2_, *L*_2_) *be two MFPs. Then, F*_1 _<<*F*_2_(*F*_1 _*is less than F*_2_) *if and only if*

((0 <*i*_2 _- *i*_1 _<*L*_1_) ∧ (*i*_2 _- *i*_1 _= *j*_2 _- *j*_1_)) ∨ ((*i*_2 _- *i*_1 _> *L*_1_) ∧ (*j*_2 _- *j*_1 _> *L*_1_))

This is a partial order relation and it can be demonstrated that the graph induced by the previous relation is a Directed Acyclic Graph (DAG). This graph can be formally described by the couple (*V*, *E*) where *V *is the set of vertices and *E *is the set of edges of the graph:

*G *≡ (*V*, *E*)   *V *= {*F *≡ (*i*, *j*, *L*) | *F *is an MFP}   *E *= {(*F*_1_, *F*_2_) | *F*_1 _<<*F*_2_}.

A path through this graph is a coherent sequence of matching pairs that can be read as an alignment between the two structures. To optimize the structural alignment, we associate a weight to every edge in the graph. An edge (*F*_1_, *F*_2_) connecting two MFPs is assigned a weight *w*(*F*_1_, *F*_2_) which is given by the sum of two terms:

*w*(*F*_1_, *F*_2_) = *w*_*F *_(*F*_1_, *F*_2_) + *w*_*C*_(*F*_1_, *F*_2_)

The first term *w*_*F*_(*F*_1_, *F*_2_) provides a measure of the local similarity of the matching pair *F*_2_. Given the measure of the distance introduced in eq. 1, we can use it to score the similarity between two fragments simply by subtracting it to the value of the *m*_*s *_threshold

*S*_*c*_(*F*_2_) = *m*_*s *_- *S*(*i*_2_, *j*_2_, *L*_2_)

This function reaches a maximum if the two fragments are exactly identical (*S*(*i*, *j*, *L*) = 0) and decreases for fragments that are increasingly different. The term *w*_*F*_(*F*_1_, *F*_2_) is calculated as the score of *F*_2 _(*S*_*c*_(*F*_2_)) multiplied by its length *L*_2_. In case of an overlap between *F*_1 _and *F*_2_, we consider only the length of the non overlapping part of *F*_2 _which is *L*_1_-*L*_2_+*i*_2_-*i*_1_

$$w_{F}(F_{1},F_{2})=\{\begin{array}{cc} L_{2}\cdot S_{c}(F_{2}) & \text{if }i_{2}-i_{1}\geq L_{1}\\ \left(L_{2}-L_{1}+i_{2}-i_{1})\cdot S_{c}(F_{2}\right) & \text{if }i_{2}-i_{1}<L_{1} \end{array}.$$

The second term, w_C_(*F*_1_, *F*_2_), is a penalization term given by the sum of two contributions: the first penalizing the presence of gaps and the second taking into account the mutual displacement of the two MFPs *F*_1 _and *F*_2 _in the two structures:

*w*_*C*_(*F*_1_, *F*_2_) = *G*_*p*_·*gap length *+ *P*(*D*_*f *_(*F*_1_, *F*_2_)).

*G*_*P *_is the gap penalty (set to -0.5 in the current implementation). The term *P*(*D*_*f*_(*F*_1_, *F*_2_)) penalizes the chaining of two MFPs that are displaced with respect to one another in the two structures.

For illustrating the function of the *P*(*D*_*f*_(*F*_1_, *F*_2_)) term, let us consider the case of an alignment including two *α*-helices. If the two helices have different relative positions in the two structures, the score for their alignment will be penalized by the *P*(*D*_*f*_(*F*_1_, *F*_2_)) term. The different relative positions can have two different reasons: Either one of the structure undergoes a conformational change moving the two helices with respect to one another (i.e. the alignment is in principle correct) or one of the two helices in one structure is in fact not structurally equivalent to its counterpart in the other structure (i.e. incorrectly aligned). In the first case, both the helices will be part of larger fragments that are structurally equivalent and the penalization introduced by the inclusion of the two helices in the alignment should be balanced by the positive contribution of the MFPs stably surrounding the two helices. If the two helices are not structurally equivalent, then the surrounding MFPs will also not be structurally equivalent thus not giving rise to balancing contributions to the score effectively leading to elimination of the two helices from the alignment.

To achieve the required behaviour of the score, *D*_*f*_(*F*_1_, *F*_2_) is defined as a measure of the displacement in space of the two matching fragments and is calculated using difference distances between the two fragments. In case the two fragments *F*_1 _≡ (*i*_1_, *j*_1_, *L*_1_) and *F*_2 _≡ (*i*_1_, *j*_1_, *L*_1_) have the same length *L *= *L*_1 _= *L*_2_, then *D*_*f *_is calculated as

$$D_{f}(F_{1},F_{2})=\frac{1}{L}\sum_{t=0}^{L-1}\left|d_{A}(i_{1}+t,i_{2}+t)-d_{B}(j_{1}+t,j_{2}+t)\right|$$

otherwise if *L *is the minimum between *L*_1 _and *L*_2 _we select in the longest fragment the subfragment of length *L *yielding to the maximum value of *D*_*f*_.

*P *is a truncated linear function calculated as

$$P(D_{f})=\{\begin{array}{cc} 0 & \text{if }D_{f}<m_{C1}\\ P_{C}\cdot L_{2}\cdot \frac{D_{f}-m_{C1}}{m_{C2}-m_{C1}} & \text{if }m_{C1}\leq D_{f}<m_{C2}\\ P_{C}\cdot L_{2} & \text{if }D_{f}\geq m_{C2} \end{array}.$$

In the current implementation the parameters are empirically set to *G*_*P *_= -0.5, P_C _= -5.0, *m*_*C*1 _= 1.0, *m*_*C*2 _= 4.0. This choice leads to preference for short gaps and longer aligned fragments with fewer hinge regions.

After weights have been assigned to all edges, the best alignment between the two structures can be seen as a *'longest path' *in the weighted graph and is calculated using a dynamic programming algorithm. Since the graph is a DAG the longest path can be calculated in time *O*(*V*+*E*) [47] where *V *is the number of MFPs and *E *is the number of edges between them. The number of edges is *O*(*V*^2^) in the worst case and the number of matching fragments is potentially *O*(*L*^2^), with *L *being the average length of the two residue sequences. This means that the worst case complexity of the overall algorithm is *O*(*L*^4^). Nevertheless, the number of matching fragments is usually much less than *L*^2 ^and several heuristics can be used to considerably speed up the algorithm.

An additional issue is taken into account while calculating the best alignment. As discussed above, a strong displacement between two MFPs is identified by a higher value of *D*_*f*_. This can happen either when the two matching fragments are located on the two sides of a hinge point or if they belong to unrelated and locally similar stretches of residues. The first case can be distinguished from the second by considering that in the case of an hinge point a pair of chained fragments with an high value of *D*_*f *_will be followed by a sequence of MFPs with lower values. Therefore correct alignments are likely to contain a lower number of chained MFPs with a high value of *D*_*f*_. Therefore, for each vertex a counter (*C*_*H*_) for the number of times the *D*_*f *_term is greater than *m*_*C*2 _on the longest path that reaches that vertex, is stored. A maximum threshold for *C*_*H *_is fixed in the algorithm (*M*_*H*_) and the algorithm discards paths leading to a value of *C*_*H *_that is higher than this threshold. In the current implementation, this threshold is fixed to 5. As a result, the alignment provided by the algorithm can cross a hinge point a number of times that must be less than *M*_*H*_. This heuristic was already used by Ye et al. [18].

### Refinement of the alignment

The initial alignment obtained after the chaining of MFPs can be used as a basis for finding additional residue equivalences that can only be detected by checking their consistency with the initial alignment.

At first, for every gap between aligned fragments, the intervening residues are systematically checked to verify if their inclusion is consistent with the rest of the alignment.

For all the aligned fragments, small shifts along the sequence (until the next aligned fragment is reached) are tested in order to correct small offsets in the alignment of periodic structures such as helices that can sometimes occur due to the high local similarity.

Finally, aligned fragments in the vicinity of the N and C-termini are inspected and eventually removed if showing insufficient quality of the alignment.

Technically, all checks are done by evaluating whether or not addition/removal of an equivalent pair of residues improves the scoring function of the genetic algorithm on the error scaled difference distance matrix between the two structures (for details on the scoring function see Schneider [29]).

### Adjustable parameters

The only adjustable parameter of the aligner is the *Low limit*. This parameter controls whether or not different distances measured between pairs of equivalent atoms are considered as identical within error. It corresponds to the *ε*_*l *_parameter in [29] and is set to 2.0 by default. The default value was optimized for the detection of typical domain motions; lower values will enforce a stricter similarity criterion for distances within rigid bodies (higher number of smaller rigid bodies) while larger values will do the opposite (leading to a lower number of rigid bodies with larger size).

### Pre-processing step

In order speed up RAPIDO for aligning structures with very similar sequences, a pre-processing step exploiting the fact that sequences can be aligned much more quickly than structures was added to the scheme described above. An initial sequence alignment is in fact performed for all pairs of structures to be aligned. If this sequence alignment reveals a sufficient similarity of the primary sequences (see below), the sequence-based equivalence map is used as a starting point for a preliminary search for rigid bodies. The rigid bodies found are retained and stored as MFPs to be later used by the RAPIDO aligner algorithm. The non-rigid and/or not aligned parts of the two structures are scanned for MFPs using the standard approach described above. The set of MFPs used for the next step of the algorithm (the merging of MFPs) is then created by combining the MFPs from the two sources.

Technically the sequence alignment is carried out using the Smith-Waterman dynamic programming algorithm [48] where a PAM250 [49] matrix is used for amino acids substitutions. If the coverage of the sequence alignment is higher than 90% or both the coverage and identity are higher than 25% the pre-processing step continues with the identification of rigid bodies, otherwise the pre-processing step is aborted and the RAPIDO algorithm is executed with no modifications.

This step is particularly useful in cases like the alignment of structures of GroEL from different organisms, where the time for computation is reduced by 80% using the pre-processing. For the human kinase dataset the pre-processing step is useful in 66% of the alignments (1496 out of 2278) and the computation time is reduced by 70% on the average.

### Compilation of the dataset of structures of protein kinase domains

All sequences of human protein kinase domains as defined in the Human Kinome database (, [50]) were used to query the database of sequences corresponding to all chains with structures deposited in the Protein Data Bank [51] with the program ssearch34 from the FASTA suite [52]. All hits with E-values less than 1*10^-90 ^were retained and manually pruned to select only structures with a sequence identity greater than 98%. With this method, for every sequence from the Human Kinome Database, all structures in the PDB that represent the respective protein were identified. For protein kinase sequences with more than one corresponding in the PDB, we then randomly selected one representative structure. The final dataset is composed of 68 structures, resulting in a total of 2278 all-against-all pairwise alignments. The PDB ids of the 68 selected structures and details about the sequence alignments are listed in additional file 3. The version of the Human Kinome database and PDB used in this study were of April 2006.

## Authors' contributions

RM conceived and implemented the aligner algorithm, compiled the hinge motions dataset, performed the tests, validated the results and drafted the manuscript. BB compiled the kinase structure dataset and validated the results on that dataset. TRS conceived, designed and coordinated the study and finalized the manuscript. All authors contributed to the discussion of the ideas behind the study. They all read and approved the final manuscript.

## Supplementary Material

Additional file 1 Comparison between DALI and RAPIDO on the human kinase structures dataset. Click here for file

Additional file 2 results on Fischer's dataset. Click here for file

Additional file 3 List of the structures included in the Dataset of human kinase structures. Click here for file
