# Supplementary material for: Alignment of protein structures in the presence of domain motions
Source: BMC Bioinformatics. 2008 Aug 27;9:352. doi: 10.1186/1471-2105-9-352 (PMC2535786; doi:10.1186/1471-2105-9-352)
Supplement: Additional file 2 — results on Fischer's dataset. [file 1471-2105-9-352-S2.doc]

**Results on Fischer’s Dataset**

Dataset taken from Fischer et al. 1996 [1]. Chain IDs where adapted from the original paper to the remediated version of the PDB (http://remediation.wwpdb.org/)

Results in the DaliLite columns where obtained using the standalone executable of DaliLite Ver. 2.4.4.

Results in the FATCAT columns where obtained directly from the original publication [2].

L1: Number of residues in the first structure

L2: Number of residues in the second structure

L: length of the alignment (number of residues aligned)

RMSD_r_: r.m.s.d. of the superimposition based on all the aligned residues (Cαs)

| **1st** | **2nd** | **L1** | **L2** | **RAPIDO** | | **DaliLite** | | **FATCAT** | |
| --- | --- | --- | --- | --- | --- | --- | --- | --- | --- |
|  |  |  |  | **L** | **RMSD_r_** | **L** | **RMSD_r_** | **L** | **RMSD_r_** |
| 1FXI_A | 1UBQ_A | 96 | 76 | 56 | 3.41 | 60 | 2.6 | 63 | 3.01 |
| 1TEN_A | 3HHR_B | 89 | 195 | 81 | 2.01 | 86 | 1.9 | 87 | 1.9 |
| 3HLA_B | 2RHE_A | 99 | 114 | 76 | 3.34 | 75 | 2.9 | 79 | 2.81 |
| 2AZA_A | 1PAZ_A | 129 | 120 | 79 | 2.43 | 81 | 2.5 | 87 | 3.01 |
| 1CEW_I | 1MOL_A | 108 | 94 | 81 | 2.37 | 81 | 2.3 | 83 | 2.44 |
| 1CID_A | 2RHE_A | 177 | 114 | 89 | 3.33 | 97 | 3.2 | 100 | 3.11 |
| 1CRL_A | 1EDE_A | 534 | 310 | 131 | 4.75 | 213 | 3.5 | 269 | 3.55 |
| 2SIM_A | 1NSB_A | 381 | 390 | 274 | 3.80 | 291 | 3.3 | 286 | 3.07 |
| 1BGE_B | 2GMF_A | 159 | 121 | 58 | 3.32 | 94 | 3.3 | 100 | 3.19 |
| 1TIE_A | 4FGF_A | 166 | 124 | 111 | 2.86 | 113 | 2.9 | 117 | 3.05 |

1. Fischer D, Elofsson A, Rice D, Eisenberg D: **Assessing the performance of fold recognition methods by means of a comprehensive benchmark**. *Pac Symp Biocomput* 1996:300-318.

2. Ye Y, Godzik A: **Flexible structure alignment by chaining aligned fragment pairs allowing twists**. *Bioinformatics* 2003, **19 Suppl 2**:II246-II255.
